# Expectancy-Value Motivation and Physical Activity- and Health-Related Outcomes among At-Risk Children and Adolescents

**DOI:** 10.3390/ijerph20136273

**Published:** 2023-07-01

**Authors:** Jiling Liu, Ping Xiang

**Affiliations:** Department of Kinesiology and Sport Management, Texas A&M University, College Station, TX 77843-4243, USA

**Keywords:** expectancy beliefs, task values, at-risk, physical activity, health

## Abstract

Despite a large amount of research having been done to examine and promote physical activity and health among adolescents and children, relatively little attention has been paid attention to underrepresented populations. In this study, we investigated the relationships between expectancy-value motivation and physical activity- and health-related outcomes among a group of at-risk boys at a summer sports camp. The total participants included 107 boys (M_age_ = 11.78 years, SD = 1.20). The boys’ perceived expectancy beliefs (EXP), importance (IMP), interest (INT), usefulness (USE), effort (EFT), and intention for future participation (IFP) were assessed using established questions on a five-point Likert scale, and a PACER test was performed to estimate their cardiovascular fitness (CVF). Through a path analysis, we found that EXP positively predicted CVF (*β* = 0.19, *p* < 0.01), IMP positively predicted EFT (*β* = 0.26, *p* < 0.01), and INT positively predicted both EFT (*β* = 0.34, *p* < 0.01) and IFP (*β* = 0.28, *p* < 0.01), while USE had no statistically significant effect on either EFT, IFP, or CVF. We discussed the limitations and implications of the present study. We recommend including a diverse sample and employing the expectancy-value model in future research, and advocating expectancy beliefs and task values, especially importance and interest, among participants during physical activity promotion.

## 1. Introduction

Regular participation in physical activity is essential to children and adolescents’ health and wellbeing. However, approximately 76% of US children and adolescents do not engage in the recommended amount of moderate-to-vigorous physical activity (MVPA) of 1 h daily [1]. Such insufficiency contributes to the obesity epidemic of childhood and adolescence, which is linked to negative health outcomes such as type 2 diabetes, heart diseases, and cancers [2]. Furthermore, ethnic and socioeconomic disparities in physical activity participation and health status have been documented [3,4]. This line of work, however, has paid little attention to a special population—at-risk children and adolescents. In this study, we focused on at-risk boys and examined their expectancy-value motivation in relation to physical activity- and health-related outcomes in a summer camp. Findings of this study may help physical educators, youth development personnel, and health professionals design effective intervention strategies to increase physical activity participation among this underrepresented group.

### 1.1. At-Risk Children and Adolescents

At-risk children and adolescents are considered those who are affected by risk factors, including poverty, insecurity, English illiteracy, and/or low parental education [5]. They are disproportionately racial and ethnic minorities, underserved, and come from low-income families. The high vulnerability of at-risk children and adolescents to these risk factors and resulting academic, social, behavioral, and health consequences have been well documented. A consensus is that at-risk children and adolescents are less likely to be physically active and more likely to have health problems. For example, disparities in MVPA participation are observed between White, Hispanic, and Black youths, with White youths having higher participation (49%) than Black (42%) and Hispanic (45%) youths [1]. The 2003 and 2007 National Survey of Children’s Health data revealed that, from 2003 to 2007, there was a 10% increase in obesity for all children and adolescents (ages 10–17) while there was a 23–33% increase for those in low education and income households and higher unemployed households [4]. Clearly, at-risk children and adolescents should be the center of concern in our efforts to address disparities in physical activity and health.

### 1.2. Expectancy-Value Motivation

To regularly participate in physical activity, children and adolescents must be motivated to do so. This is because motivation is a driving force for people to initiate and sustain effort toward desired outcomes [5]. In both theoretical and empirical works, expectancy-value motivation has been found to be significantly related to children and adolescents’ affective, cognitive, and behavioral outcomes in a variety of settings, including physical activity/physical education [6].

The expectancy-value model consists of two components: expectancy beliefs and task values [7,8]. Expectancy beliefs refer to how capable they believe themselves to be in succeeding at a task, and task values are defined as the degree to which they value the activity. Task values consist of four subcomponents: attainment value or importance, intrinsic value or interest, utility value or usefulness, and cost. Because the first three task values are the most researched in the literature, we focused on them in the present study. Importance deals with beliefs about the importance of doing well on a given task, interest refers to the enjoyment derived from engaging in the task, and usefulness concerns how useful the performer perceives the task undertaken. Compared to importance and interest, which are more intrinsic to the performer, usefulness is more of extrinsic motivation [8].

The literature shows that children and adolescents’ expectancy-value motivation (expectancy beliefs and task values) is significantly related to important outcomes in physical activity/physical education settings, such as physical activity engagement, effort and persistence, task performance, and intention for future physical activity participation [6]. For example, Xiang and colleagues [9] reported that intrinsic and importance values positively predicted fourth graders’ intentions for future participation in the physical education of running, while expectancy beliefs positively predicted their performance of a 1-mile run test. Chen and Chen [10] observed that ninth graders who had a higher level of expectancy beliefs and intrinsic value tended to be more physically active in physical education classes. A positive association between expectancy beliefs and after-school physical activity participation also emerged in a sample of physical education students in grades three to five [11]. However, little work has been conducted to examine whether the relationships between expectancy-value motivation and physical activity-related outcomes observed with general populations also exist among at-risk children and adolescents. In this study, we specifically focused on the three outcomes that most often appeared in the literature, namely, effort, intention for future physical activity participation, and cardiovascular fitness, and described them below.

### 1.3. Effort, Intention for Future Physical Activity Participation, and Cardiovascular Fitness

To benefit from regular physical activity, children and adolescents need to invest effort. Effort refers to the overall amount of energy spent during physical activity participation. It is an outcome highly valued in our society. Research indicates that students who are willing to put forth great effort or work hard tend to perform well, achieve success, and benefit most from participating in physical activity/physical education [12,13].

Another outcome critical to our understanding of physical activity participation among at-risk children and adolescents is their intention to participate in physical activity in the future, on the grounds that individuals’ behavioral intentions are highly related to actual achievement behaviors [14]. For example, Xiang et al. [15] reported that, across the fifth- and sixth-grade data, students’ intention for future participation in running was significantly correlated with their 1-mile run test performance. That is, students with a stronger intention to participate in future running activities completed the 1-mile run test in less time than their peers who did not have such a strong intention.

Finally, cardiovascular fitness is considered an important predictor of physical and mental health in children and adolescents. It refers to the overall aerobic capacity of the cardiovascular, respiratory, and muscular systems during extended physical activity participation. High cardiovascular fitness tends to result in multiple benefits, including lower risk for cardiovascular disease, improved academic performance, and higher levels of psychological well-being [16,17]. However, there is little empirical evidence for the effect of expectancy-value motivation on this important health indicator. Given the significance of cardiovascular fitness and research vacancy, it is important for us to examine how expectancy-value motivation would be associated with cardiovascular fitness among at-risk adolescent boys in the present study.

### 1.4. The Present Study

Based on the reasons stated above, the purpose of the present study was to examine the relationships between expectancy-value motivation and physical activity- and health-related outcomes among a group of at-risk children and adolescents. Identifying the relationships between these physical activity- and health-related variables not only adds to the body of knowledge about research on underserved populations, but also provides an expanded understanding of what is behind participants’ health behavior choices and outcomes, as well as informs physical educators, youth development personnel, and health service providers about how to design and deliver effective intervention strategies, especially for underserved populations, to address health disparities.

In the present study, we specifically focused on the following three research questions: (1) How would the components of expectancy-valve motivation (i.e., expectancy beliefs, importance, interest, and usefulness) each predict the participants’ effort during their physical activity sessions? (2) How would each expectancy-value motivation component predict their intention for future physical activity participation after the participants’ camp time? And (3) how would each expectancy-value motivation component predict the participants’ cardiovascular fitness levels at the end of the camp time? Based on the theorization of expectancy-value motivation and the literature, we hypothesized that each component of expectancy-value motivation would positively predict the participants’ effort, intention for future physical activity participation, and cardiovascular fitness.

## 2. Materials and Methods

### 2.1. Participants and Setting

A total of 110 at-risk boys volunteered for this study. However, due to uncompleted questionnaires, 107 boys (M_age_ = 11.78 years, SD = 1.20) were retained in our dataset. Among these participants, 20 (18.69%) were White, 19 (17.76%) Black, 63 (58.88%) Hispanic, and 5 (4.67%) Others (e.g., mixed racial or unidentified). At the time of the study, they were enrolled in a cost-free, overnight summer sports camp in the Southwest. To be admitted to the sports camp, boys must meet a few criteria, such as being associated with at least one risk indicator (e.g., on the National School Lunch Program, lower household income, lower parent education), aged 10–13, and permitted by parents. Note that the camp does not admit girls due to operational efficiency concerns. After admission, the camp director assigns 7 boys to a cabin that is supervised by a coach, and the coach and the boys will live camp life together for the next three weeks. All coaches have at least one year of teaching or coaching experience before they are hired. Through living and having activities together, the coach teaches the boys valuable character assets, including discipline, honesty, respect, and collaboration. The camp offers a variety of physical activities such as track and field, basketball, baseball, archery, and canoeing. During a typical day, coaches will organize and lead activities. The boys are also scheduled to have a few field trips, including a visit to a nearby university campus.

### 2.2. Variables and Measures

Participants’ demographic information, such as age and ethnicity, was collected with a personal data sheet. All variables were measured with previously validated questionnaires [18,19].

#### 2.2.1. Independent Variables

(1) Expectancy Beliefs. Five questions were used to assess the participants’ expectancy beliefs toward the camp activities. They are (1) “How good are you at the activities you did in these sessions?”, (2) “If you were to list all the students in these sessions from the worst to the best, where would you put yourself?”, (3) “Some kids are better in one activity than in another. For example, you might be better in basketball dribbling than in soccer kicking. Compared to most of your other camp activities you do, how good are you at the activities you learned in these sessions?”, (4) “How well do you think you learned the activities in these sessions this summer?”, and (5) “How good were you at learning new physical activities in these sessions?” Boys rated each question on a 5-point Likert scale from 1 = “*Very bad*” to 5 = “*Very good*”.

(2) Importance. Two questions were used to assess the participant’s perceptions of the importance of participating in the camp activities: (1) “For me, being good at activities in these sessions is” and (2) “Compared to other camp activities, how important is it for you to be good at the physical activities in these sessions?” Boys responded to the questions on a 5-point Likert scale (1 = “*Not very important*”, and 5 = “*Very important*”).

(3) Interest. Two questions were used to assess the participant’s interest in participating in the camp activities. Boys responded to the first question, “In general, I found learning new activities in these sessions”, on a 5-point Likert scale from 1 = “*Very boring*” to 5 = “*Very fun*”. They rated the second question, “How much do you like the activities you learned in these sessions?” with 1 = “*Didn’t like it at all*”, and 5 = “*Liked it very much*”.

(4) Usefulness. Two questions were used to assess how the participants felt the camp activities were useful: (1) “Some things that you learned in school help you do things better outside of class. We call this being useful. For example, learning about plants might help you grow a garden. In general, how useful are the activities that you learned in these sessions?” and (2) “Compared to other camp activities, how useful are the activities you learned in these sessions?” Boys responded to the questions on a 5-point Likert scale (1 = “*Not at all useful*”, and 5 = “*Very useful*”).

#### 2.2.2. Dependent Variables

(1) Effort. With a leading question stem, “I participate in camp physical activities because”, four questions were used to assess the participants’ effort put into the camp activities, including (1) “I worked hard to do well even if I did not like something we are doing”, (2) “I spent extra time and effort trying to do well”, (3) “I overcame difficulties to participate every day”, and (4) “I pushed myself as far as possible when I was already physically tired”. Boys responded to the questions on a 5-point Likert scale (1 = “*Not true for me*”, and 5 = “*Very true for me*”).

(2) Intention for future physical activity participation. With a leading question stem, “Right after this camp is over, during my leisure time I”, three questions were used to assess the participants’ intention for future physical activity participation after the camp. They are: (1) “Decided to do physical activity that makes me breathe hard or feel tired”, (2) “Will try to do physical activity that makes me breathe hard or feel tired”, and (3) “Plan to do physical activity that makes me breathe hard or feel tired”. Boys responded to the questions on a 5-point Likert scale (1 = “*Not true for me*”, and 5 = “*Very true for me*”).

(3) Cardiovascular fitness. Because of its adequate reliability and validity [20], the Progressive Aerobic Cardiovascular Endurance Run Test (PACER) [21] test was used to assess the participants’ cardiovascular fitness level. Following the FITNESSGRAM Reference Guide [22], the participants ran progressive 20 m laps between two lines, and the max number of laps that a participant could run was recorded to indicate their cardiovascular fitness level.

### 2.3. Data Collection and Analysis

Prior to data collection, we obtained the university Institutional Review Board approval, parent consent, and minor assent. During the last week of the camp, we administered the personal data sheet and questionnaires during a lunchtime. We allowed 25 min for the participants to ask questions and complete all questionnaires. Later in the same day during the afternoon activity session, we and trained camp coaches administered the PACER test and asked the boys to run as best as they could, and five boys were assessed each time This data collection was completed in about one hour.

Data analyses were done using SPSS (Version 23.0, IBM Corp., Armonk, NY, USA) and Mplus (Version 7.4, Muthén & Muthén, Los Angeles, CA, USA). We performed a data screening before the analyses. Uncompleted questionnaires were removed, and missing values and outliers computed. Then, we verified construct validity for the expectancy-value scales and the effort scale. Because the intention for future physical activity participation scale has three items only, it will always result in a perfect model fit, and it is not necessary to verify its construct validity. Next, we calculated descriptive statistics (e.g., Means, SD) and scale reliability (Cronbach’s *α*) for all variables. After, we computed correlations to identify bivariate relationships. Finally, to gain a wholistic view of the relationships between the variables, we conducted a path analysis aimed to reveal how the participants’ expectancy beliefs, importance, interest, usefulness, would predict their effort, intention for future physical activity participation, and cardiovascular fitness. The global model fit indices used for the construct validity tests and the path analysis included the Chi-Square test (*χ*^2^), Root Mean Square Error of Approximation (RMSEA), Comparative Fit Index (CFI), and Standardized Root Mean Square Residual (SRMR). When *p* > 0.05 for the *χ*^2^ test, this indicates the tested model fits the data well [23]. At the same time, when RMSEA ≤ 0.08, CFI < 0.90, and SRMR < 0.08, the model fit is acceptable [24].

## 3. Results

### 3.1. Construct Validity of Measurement

Our construct validity test for the expectancy-value motivation scales resulted in an acceptable model fit, *χ*^2^_(37)_ = 49.10, *p* = 0.09; RMSEA = 0.05; CFI = 0.97; SRMR = 0.05. The four-item effort scale had acceptable construct validity as well, *χ*^2^_(2)_ = 1.10, *p* = 0.58; RMSEA = 0.00; CFI = 1.00; SRMR = 0.02. These results demonstrate that the scales used to assess the variables were valid for the present study. Based on the validity results, we proceeded to the next step of data analysis.

### 3.2. Descriptive Statistics and Scale Reliability

Table 1 shows Mean, standard deviation, Skewness, Kurtosis, and Cronbach’s *α* values for all variables. The participants scored the six five-point Likert scales all above the mid-point of three. These mean scores indicate that their interest (INT) in the camp physical activities was relatively higher, while intention for future participation (IFP) in physical activity after the camp remained relatively lower than all the other variables. The average laps the participants ran was 20 and a half. All Skewness and Kurtosis values range between −1 and +1, indicating the data were approximately normally distributed [25]. All Cronbach’s *α* values are above 0.60, showing that these scales had an acceptable internal consistency [26].

### 3.3. Bivariate Correlations

Bivariate correlations between all variables are presented in Table 2. It shows that all variables from the expectancy-value model components are moderately positively correlated, as its theorization proposes [7,8]. The outcome variables, particularly effort (EFT) and IFP, are also positively correlated with all expectancy-value constructs. Cardiovascular fitness (CVF) is positively correlated with expectancy beliefs (EXP), INT, usefulness (USE), and IFP, but not significantly correlated with importance (IMP) and EFT.

### 3.4. Path Analysis

**Figure 1:**
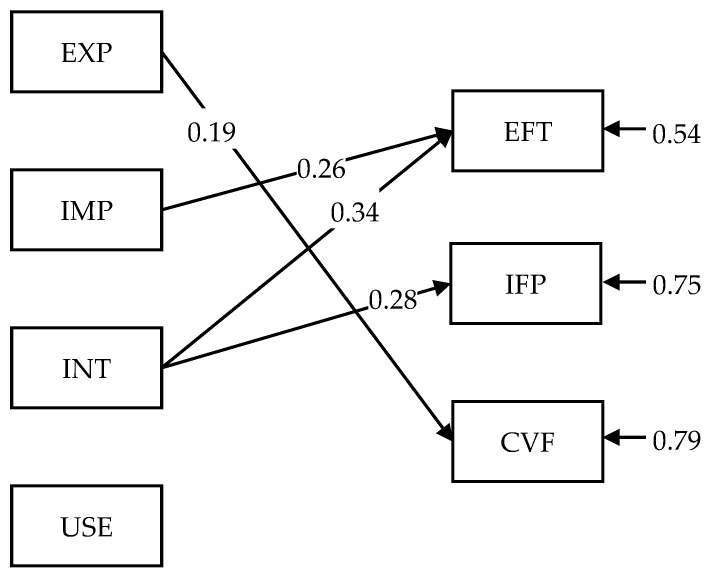
Path analysis. Only statistically significant (*p* < 0.05) paths are shown.

To answer the three research questions, we conducted a path analysis with a robust maximum likelihood estimator. In this model, EXP, IMP, INT, and USE are predictors, while EFT, IFP, and CVF are outcome variables. The path model generated a perfect model fit. Figure 1 shows EXP had a significant, positive effect (*β* = 0.19, *p* < 0.01) on CVF, IMP had a significant, positive effect (*β* = 0.26, *p* < 0.01) on EFT, and INT positively predicted both EFT (*β* = 0.34, *p* < 0.01) and IFP (*β* = 0.28, *p* < 0.01), while USE had no statistically significant effect on any of the outcome variables. The R-square values explained in this model for EFT, IFP, and CVF are 0.46, 0.25, and 0.21, respectively, and these values are all statistically significant (*p* < 0.01).

## 4. Discussion

Regular physical activity participation and health are critical topics among children and adolescents. Although there is plenty of research on these two topics, studies devoted to underrepresented populations are scarce. Especially rare are such investigations through the lens of the expectancy-value model [7,8]. To address these concerns, we examined how expectancy-value motivation would affect physical activity- and health-related outcomes among a group of at-risk boys in this study. Overall, we found expectancy beliefs, importance, and interest emerged as significant predictors to respective outcome variables. The results partially supported our hypothesis that the components of expectancy-value motivation would each positively predict the boys’ effort, intention for future physical activity participation, and cardiovascular fitness.

Research question 1 asks how the components of expectance-value motivation would each predict the participants’ effort during their physical activity sessions in the sports camp. Consistent with Gao et al. [27] but contrary to Cox and Whaley [28] and Xiang, McBride and Bruene [18], we did not find the boys’ expectancy beliefs to have any effect on effort. Just as in the three studies [18,27,28], our study did not find the predictability of usefulness to effort among these boys, either. Partially supporting our hypothesis and consistent with the theorization [7,8], the boys who valued their activities as important and interesting invested effort during camp activities. This result, however, is contradictory to what was found in the literature—Cox and Whaley [28], Xiang, McBride and Bruene [18], and Gao [19]. Neither of these studies identified a statistically significant effect from importance or interest to effort. This might be due to different groups being sampled in these studies. In Cox and Whaley [28], participants were high school varsity basketball players, while in Xiang, McBride and Bruene [18] and Gao [19], participants ranged from kindergarteners to middle schoolers. In the present study, participants were exclusively underserved boys. Facing risks factors at school and home, they might deem the variety of physical activities offered in the sports camp especially important and interesting, and therefore were willing to actively take part in them. Further studies with qualitative design, however, may be done to verify this assumption.

Regarding research question 2, we asked if the components of expectancy-value motivation would each predict participants’ intention for future physical activity participation after their camp time. Consistent with Xiang et al. [9,18] and Gao and Xiang [29] but different from Gao, Lodewyk and Zhang [27], our study did not find the boys’ expectancy beliefs or usefulness predicted their intention for future participation. Our study also confirmed Xiang et al. [9,18] in that importance did not predict intention for future participation, but this is not the case in Gao and Xiang [29] and Gao, Lodewyk and Zhang [27]. Again, these mixed results might be attributed to different samples and different settings. As explained before, our sample was exclusively at-risk boys, while samples from other studies were all school students including both genders and with various backgrounds. Residing in a structured sports camp may induce a different experience from naturally attending a public school [27,29]. Hence, further studies based on the expectancy-value model may provide a clearer view of the relationships between these components and intention for future physical activity participation. Corroborating the literature [9,18,27,29], our study revealed that interest also emerged as a single significant predictor of intention for future physical activity participation. This result informs health professionals interested in addressing physical activity-related health disparities among children and adolescents that, regardless of participant characteristics and settings, a higher level of interest can always lead to a stronger intention for future participation in physical activity. When examining the relationships among motivation, physical activity, and health outcomes, researchers may as well consider including this significant variable (i.e., interest) in their studies.

Our results concerning research question 3 partially support our hypothesis, in that only expectancy beliefs positively predicted cardiovascular fitness, while task values did not affect this outcome variable. Nevertheless, this finding is entirely aligned with the literature (e.g., [29,30]). Particularly, Gao [19], Gao, Lodewyk and Zhang [27], and Zhu and Chen [30] used the PACER test as the indicator of participants’ cardiovascular fitness, and they all found expectancy beliefs positively predicted PACER test results. This is identical to our finding that at-risk boys with higher expectancy beliefs tended to run more laps. Because the PACER test is a performance-related task, it is understandable that when feeling capable and competent, the boys tended to demonstrate their capability and competence as well as their desire to succeed during the PACER test. This predictability of expectancy beliefs also holds true to other performance-related tasks such as running [9,18] and weightlifting [29]. When it comes to task values, however, these variables do not seem to affect task performance at all. Our study, together with other empirical studies [18,19,30], found that neither importance, interest, nor usefulness had any effect on cardiovascular fitness. This may be traced to the theorization of the expectancy-value model [7,8], where task values were conceptualized to influence individuals’ choice of behavior rather than task performance. Note that, although often correlated with most variables, usefulness has consistently been found to be a non-predictor for physical activity- and health-related outcomes. Particularly in our study, at-risk children and adolescents seemed not to perceive how the PACER test processes utility values as indicators of their effort or cardiovascular fitness. Moreover, because usefulness is often associated with extrinsic motivation [8], physical educators, youth development personnel, and health service providers are not suggested as promoting the utility values of physical activity among participants.

Although the present study provides a unique perspective of the relationships among expectancy-value motivation and physical activity- and health-related outcomes among an underrepresented population, there are a few areas of concern we would like to acknowledge before generalizing our findings. First, our sample only includes at-risk boys. When comparing our results to those of other studies, we caution scholars to consider gender differences and participants’ background information (e.g., socioeconomic status). For future research, we suggest including both boys and girls to make a better representation of underserved populations. Second, the subjective, self-rated responses to the questionnaire may not truthfully reflect the participants’ effort. For further studies, assessment tools such as accelerometers and ergometers may be used to provide more objective and accurate measurement. At the same time, we noticed that there is still a dearth of studies based on the expectancy-value model within the field of physical education/physical activity and sports. Therefore, we call for more attempts to employ the expectancy-value model to guide future research, so that we may have a more comprehensive understanding of how each component of the model works for physical activity- and health-related outcomes. So doing may enhance the efficacy of our effort to address physical activity-related health disparities in children and adolescents.

Despite these limitations, the results of our study still offer insightful implications for practice. Based on the positive relationships between expectancy beliefs and health-related outcomes in our study, and the previous studies, it seems always conducive to strengthen participants’ expectancy beliefs when completing performance-related tasks. Therefore, for this particular camp, we recommend coaches to provide at-risk boys with encouragement, positive feedback, and inclusive activities for everyone to feel supported and experience success. Regarding task values, importance and interest should be emphasized. Camp coaches can explain to the boys why an activity is important and the benefits of participation before starting an activity session. This way, these boys may be more willing to to engage in the activity. Making activities fun and interesting is critical to the boys’ intrinsic desire of continued physical activity even after their current participation, which, in the long run, can lead to a physically active and healthy lifestyle. When it comes to similar physical activity promotion or intervention settings, physical educators, youth development personnel, and health service providers may follow the same recommendations to make their promotion/intervention more effective. In addition, we reserve our recommendations on advocating usefulness, because of its association with extrinsic motivation and its always being an ineffective motivating factor across studies, including the present study.

## 5. Conclusions

The present study serves as the first attempt at utilizing the expectancy-value model to respond to the health disparity issues among underrepresented youth. Through examining the relationships between expectancy-value motivation and physical activity- and health-related outcomes among a group of at-risk boys at a summer sports camp, we discussed implications for future research and practice. In addition, our study provides initial evidence for the psychometric acceptability in underserved youth within a physical activity setting.

## Figures and Tables

**Table 1:** Descriptive statistics and scale reliability (N = 107).

	Mean	SD	Skewness	Kurtosis	*α*
EXP	3.90	0.60	0.15	−0.83	0.83
IMP	4.03	0.69	−0.32	−0.35	0.65
INT	4.34	0.55	−0.43	−0.45	0.85
USE	4.11	0.61	−0.33	−0.78	0.68
EFT	4.11	0.61	−0.33	−0.78	0.76
IFP	3.61	1.23	−0.74	−0.44	0.95
CVF	20.49	11.33	1.00	0.60	0.83

Note. EXP = expectancy beliefs, IMP = importance, INT = interest, USE = usefulness, EFT = effort, IFP = intention for future physical activity participation, CVF = cardiovascular fitness.

**Table 2:** Correlations between all variables (N = 107).

	EXP	IMP	INT	USE	EFT	IFP	CVF
EXP	--						
IMP	0.51 **	--					
INT	0.54 **	0.56 **	--				
USE	0.58 **	0.49 **	0.56 **	--			
EFT	0.41 **	0.44 **	0.50 **	0.37 **	--		
IFP	0.34 **	0.35 **	0.38 **	0.37 **	0.36 **	--	
CVF	0.31 **	0.04	0.24 *	0.25 *	0.12	0.26 **	--

** *p* < 0.01, * *p* < 0.05.

## Data Availability

The data presented in this study are available upon request from the corresponding author. The data are not publicly available due to participant privacy.
